# Investigating the lesion detectability of Tc‐99m planar scintigraphy acquired with LEHRS collimator for patients with different body sizes: A phantom study

**DOI:** 10.1002/acm2.13744

**Published:** 2022-08-10

**Authors:** Pei‐Yao Lin, Kai‐Jie Jhan, Kuan‐Yin Ko, Ching‐Ching Yang

**Affiliations:** ^1^ Department of Nuclear Medicine National Taiwan University Cancer Center Taipei Taiwan, ROC; ^2^ Department of Nuclear Medicine National Yang‐Ming University Hospital Yilan Taiwan, ROC; ^3^ Department of Medical Imaging and Radiological Sciences Kaohsiung Medical University Kaohsiung Taiwan, ROC; ^4^ Department of Medical Research Kaohsiung Medical University Chung‐Ho Memorial Hospital Kaohsiung Taiwan, ROC; ^5^ Graduate Institute of Clinical Medicine, College of Medicine National Taiwan University Taipei Taiwan. ROC

**Keywords:** body size, Clarity 2D blending weight, LEHRS collimator, lesion detectability, Tc‐99m planar scintigraphy

## Abstract

**Purpose:**

The aim of this work was to investigate the lesion detectability of Tc‐99m planar scintigraphy acquired with a low‐energy high‐resolution and sensitivity (LEHRS) collimator and processed by Clarity 2D for patients with different body sizes through phantom study.

**Methods:**

A NEMA IEC body phantom set was covered by two layers of 25‐mm‐thick bolus to construct phantom in three different sizes. All image data were performed on a Discovery NM/CT 870 DR with an LEHRS collimator and processed by Clarity 2D with blend ratio a of 0%, 20%, 40%, 60%, 80%, and 100%. The lesion detectability in gamma scintigraphy was evaluated by calculating the contrast‐to‐noise ratio (CNR). Multiple linear regression methods were used to analyze the impact of body size, target size, and Clarity 2D blending weight on the lesion detectability of Tc‐99m planar scintigraphy.

**Results:**

It was found that changing the blend ratio could improve CNR, and this phenomenon was more significant in anterior view than in posterior view. Our results also suggested that the blend ratio should be selected according to patient body size in order to maintain consistent CNR. Hence, when a blend ratio of 60% was used for a patient before cancer treatment, a lower blend ratio should be used for the same patient experiencing treatment‐related weight loss to achieve consistent lesion detectability in Tc‐99m planar scintigraphy acquired with LEHRS and processed by Clarity 2D.

**Conclusion:**

The magnitude of photon attenuation and scattering is higher in patients with larger body size, so Tc‐99m planar scintigraphy usually has lower lesion detectability in obese patients. Although photon attenuation and scattering are inevitable during image formation, their impacts on image quality can be eased by employing appropriate image protocol parameters.

## 1 INTRODUCTION

Tc‐99m is the most used radionuclide in nuclear medicine imaging because it fulfills many of the criteria of an ideal radionuclide, such as half‐life, decay mode, and photon energy.^1^, ^2^, ^3^ In our routine practice, scintigraphic exams using Tc‐99m radiolabeled pharmaceuticals are usually acquired with a low‐energy high‐resolution (LEHR) collimator.^4^, ^5^ Collimator design is a compromise between spatial resolution and sensitivity.^6^, ^7^ There are three common designs for a parallel hole collimator, including high resolution, general purpose, and high sensitivity. Among the three collimator designs, a high resolution collimator has the worst sensitivity, whereas a high sensitivity collimator has the worst spatial resolution.^8^, ^9^ The spatial resolution and system sensitivity of a Discovery NM/CT 870 DR with LEHR collimator (GE Healthcare, Milwaukee, WI, USA) for Tc‐99m were 7.4 mm and 72 cps/MBq, respectively. The GE LEHR collimator has a hole diameter of 1.5 mm, a septal thickness of 0.2 mm, and a hole length of 35 mm. A novel LEHR and sensitivity (LEHRS) collimator designed by GE Healthcare has a hole diameter of 1.43 mm, a septal thickness of 0.13 mm, and a hole length of 32 mm. The spatial resolution and system sensitivity of a Discovery NM/CT 870 DR with LEHRS collimator (GE Healthcare, Milwaukee, WI, USA) for Tc‐99m were 7.4 mm and 92 cps/MBq, respectively. Moreover, the planar scintigraphy acquired with the LEHRS collimator can be further processed by a software called Clarity 2D (GE Healthcare, Milwaukee, WI, USA) to reduce noise and improve contrast. Hence, the lesion detectability of gamma scintigraphy acquired with LEHRS collimator is better than that acquired with the conventional LEHR collimator, which has been reported by previous studies.^10^, ^11^ However, photon attenuation and scattering are more likely to occur in obese patients compared to skinny patients, hence degrading the lesion detectability of gamma scintigraphy more seriously.^12^, ^13^ To the best of our knowledge, the impact of body size on Tc‐99m planar scintigraphy acquired with the newly introduced LEHRS collimator has not been investigated yet. Therefore, this study aimed to assess lesion detectability in NEMA IEC body phantom using Tc‐99m planar scintigraphy acquired with an LEHRS collimator and Clarity 2D image processing. The simultaneous effects of different body sizes, lesion diameters, and Clarity blending weights on lesion detectability, quantified by contrast‐to‐noise ratio (CNR), were investigated by using multivariate analysis.

## 2 METHODS

### 2.1 Phantom design

**FIGURE 1:**
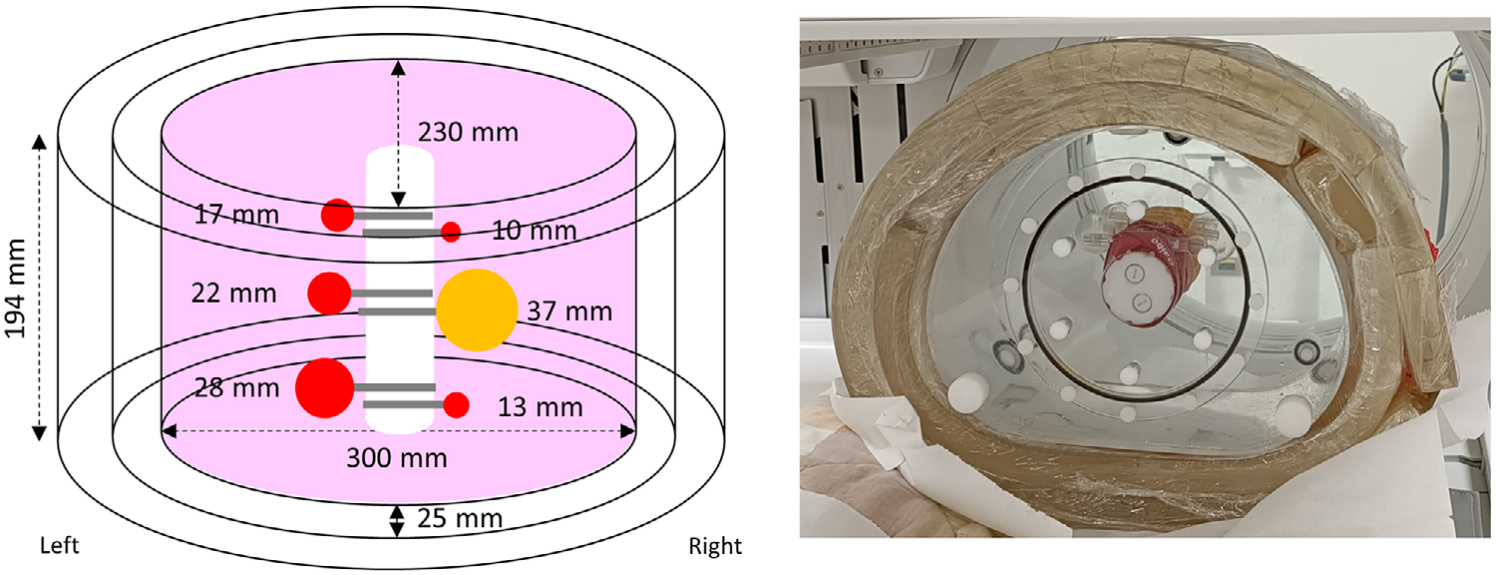
Illustration (left) and photo (right) of the NEMA IEC phantom

The NEMA IEC body phantom set (Capintec, Florham Park, NJ, USA), which consists of a body phantom, a lung insert and six spheres with diameters of 10, 13, 17, 22, 28, and 37 mm, was used in this study (Figure 1). The outer dimensions of the phantom are 300 × 230 mm^2^ in a transverse plane and 194 mm in height (NEMA_small_). The body phantom was covered by two layers of 25‐mm‐thick bolus (Superflab Bolus; Radiation Products Design Inc, Albertville, MN, USA) to enlarge the phantom size in transverse plane to 350 × 280 (NEMA_medium_) and 400 × 330 mm^2^ (NEMA_large_). The lung insert is a cylinder filled with water and the molded expanded polystyrene beads to simulate lung density. The spheres with diameters of 10, 13, 17, 22, and 28 mm were filled with a Tc‐99m solution of 300 kBq/ml to simulate tumor, and the 37‐mm‐diameter sphere was filled with Tc‐99m solution of 50 kBq/ml to simulate normal tissues. The body phantom was filled with a Tc‐99m solution of 8 kBq/ml to simulate background uptake. Table 1 summarizes the characteristics of the NEMA IEC phantom used in this study. As shown in Figure 1, the stems of the six spheres were attached to the lung insert to prevent overlapping between spheres when acquiring gamma scintigraphy.

**TABLE 1:** The characteristics of the NEMA IEC phantom

Compartment	Volume (ml)	Activity concentration (kBq/ml)	Activity (kBq)
10‐mm sphere	0.52	300	156
13‐mm sphere	1.14	300	342
17‐mm sphere	2.57	300	771
22‐mm sphere	5.57	300	1671
28‐mm sphere	11.49	300	3447
37‐mm sphere	26.52	50	1326
Lung insert	194	0	0
Torso cavity	9700	8	77 600

### 2.2 Data acquisition

All image data were performed on a Discovery NM/CT 870 DR with an LEHRS collimator (GE Healthcare, Milwaukee, WI, USA). The planar scans were acquired with 140.5 keV ±7.5% of photo‐peak window width, 256 × 256 matrices, 2.2 mm of pixel size. Acquisitions were obtained for a minimum of 500 000 counts in the anterior and posterior views. For static scan and whole‐body scan acquired with an LEHRS collimator, Clarity 2D (GE Healthcare, Milwaukee, WI, USA) can be used to process the image data, which incorporates three procedures, including (1) noise reduction, (2) contrast enhancement, and (3) blending against planar or whole‐body images. In Clarity 2D processing, noise reduction was conducted through iterative edge preserving filtering with an adaptive bilateral filter,^14^, ^15^ whereas contrast enhancement was conducted by the Lucy–Richardson deconvolution with Laplace/Gauss kernel determined empirically.^16^, ^17^ As for the blending step, the original image (*I*_original_) and the processed image (*I*_processed_) were mixed according to the following equation:

(1) $$I=\left(1-\mathrm{blend}\;\mathrm{ratio}\right)\times I_{\mathrm{original}}+\left(\mathrm{blend}\;\mathrm{ratio}\right)\times I_{\mathrm{processed}}$$

By default, the blend ratio was set as 40%. In this study, the gamma scintigraphy for each phantom size was processed by using a blend ratio of 0%, 20%, 40%, 60%, 80%, and 100%.

### 2.3 Image quality assessment

**FIGURE 2:**
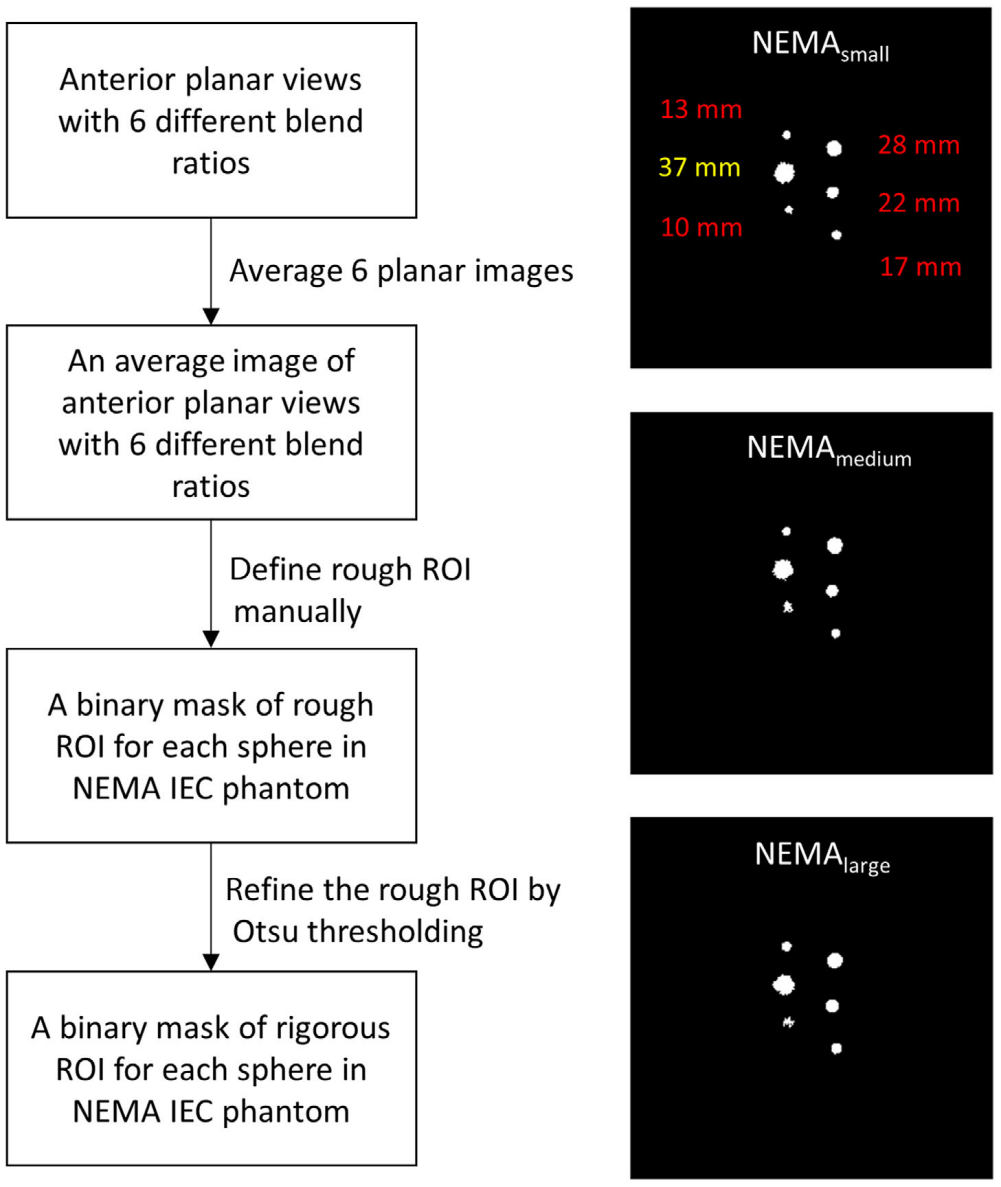
The flowchart of region‐of‐interest (ROI) determination (left) and the resulting binary masks for six spheres in NEMA_small_, NEMA_medium_, and NEMA_large_ (right)

Figure 2 illustrates the flowchart to determine the region of interest (ROI) for the spheres in NEMA IEC phantom. First, six anterior planar views with different blend ratios were averaged. Next, an ROI containing hot sphere and some part of background was drawn manually on the averaged image for each sphere (i.e., the rough ROI). The Otsu thresholding was then used to refine the ROI by differentiating the sphere and background pixels (i.e., the rigorous ROI). The binary masks for the spheres in NEMA_small_, NEMA_medium_, and NEMA_large_ generated through the proposed workflow were also shown in Figure 2. The mean and standard deviation within the rigorous ROIs were then calculated for both anterior and posterior views.

The lesion detectability in gamma scintigraphy was evaluated by calculating CNR, which was defined as

(2) $$\mathrm{CNR}\;=\frac{{\mathrm{AVG}}_{\mathrm{tumor}}\;-\;\;\mathrm{AV}G_{\mathrm{normal}}}{{\mathrm{SD}}_{\mathrm{normal}}}$$

AVG_tumor_ is the mean counts of the spheres simulating tumor. AVG_normal_ and SD_normal_ are the mean and standard deviation of photon counts of the 37‐mm‐diameter sphere, respectively. A CNR of 1.0 occurs when the image contrast (or difference) between tumor and normal tissue was equal to the statistical fluctuation in normal tissues.

### 2.4 Multivariate analysis

Multiple linear regression methods were used to analyze the impact of (1) body size, (2) target size, and (3) Clarity 2D blending weight on the lesion detectability, which was quantified as CNR, of Tc‐99m planar scintigraphy acquired with an LEHRS collimator and processed by Clarity 2D. Ordinary least squares regression was used to predict the dependent variable from the independent variables. The model to explain the dependence relationship was defined as

(3) $$\begin{array}{ccc} \mathrm{CNR} & = & gB_{0}+B_{1}\times \left(\frac{1}{\mathrm{body}\;\mathrm{size}}\right)+B_{2}\times \left(\mathrm{target}\;\mathrm{size}\right)\\ & & +\;B_{3}\times \left(\log\left(1+\mathrm{blend}\;\mathrm{ratio}\right)\right) \end{array}$$

where *B*_0_ to *B*_3_ were the regression coefficient (*B*) to be estimated. Body size was the square root of the product of long‐ and short‐axis of the phantom (i.e., the effective body diameter). Target size was the diameter of spheres in NEMA IEC phantom. The standard regression coefficient (*β*) was calculated to assess the relative importance of each predictor. Student's *t* test and variance inflation factor (VIF) were used as criteria in screening the potential regression model. A predictor was considered statistically significant if |*t*| > 2. A maximal VIF value in excess of 10 was regarded as an indication that multicollinearity may be unduly influencing the least‐square estimates. The coefficient of determination (*R* ^2^) was calculated to assess the strength of the functional regression model. The statistical analysis algorithms were implemented in MATLAB 7.1 (The MathWorks, Natick, MA, USA).

## 3 RESULTS

**FIGURE 3:**
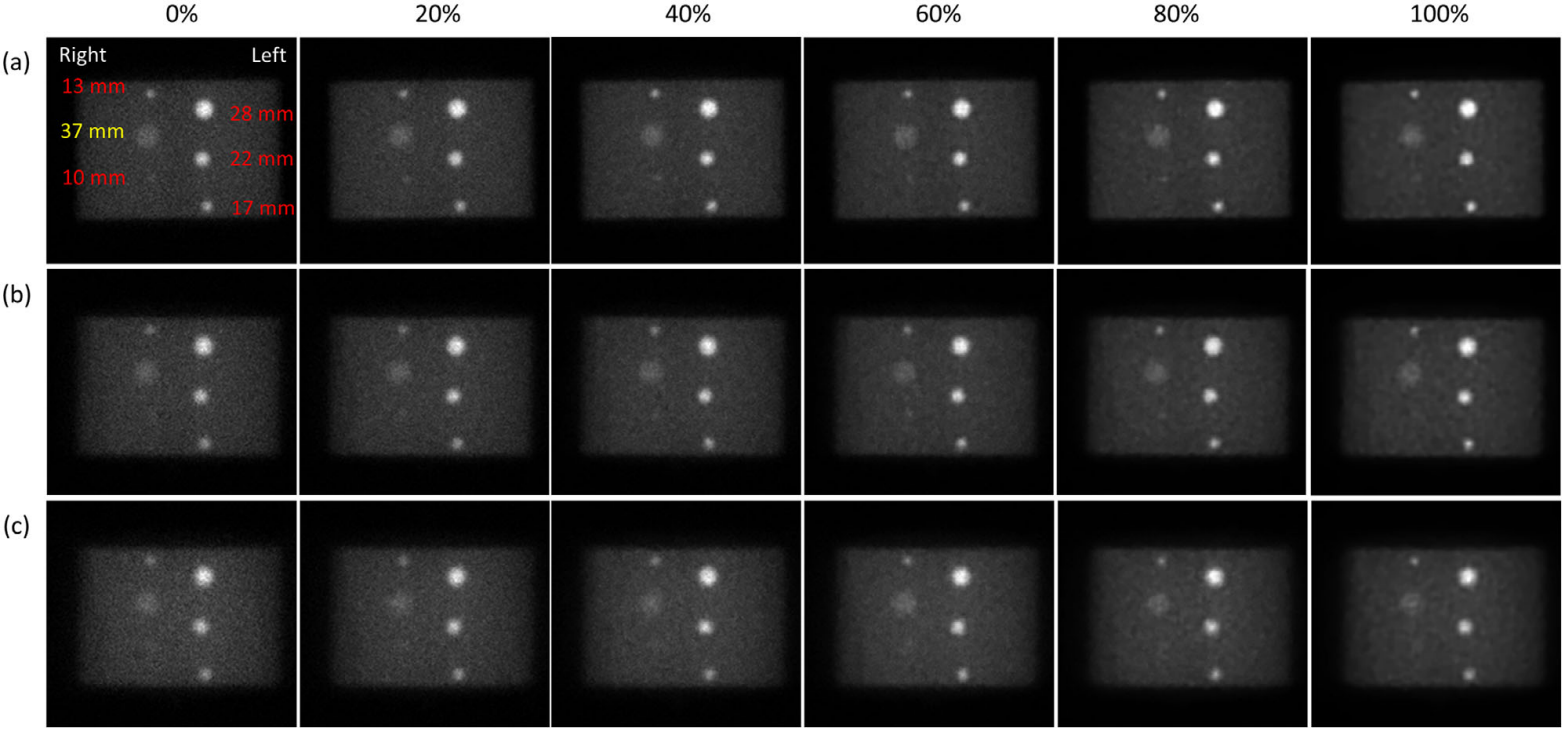
Anterior view of Tc‐99m planar scintigraphy (window level = 100/window width = 200) acquired with a blend ratio of 0%, 20%, 40%, 60%, 80%, and 100% (from left to right) for (a) NEMA_small_, (b) NEMA_medium_, and (C) NEMA_large_

**FIGURE 4:**
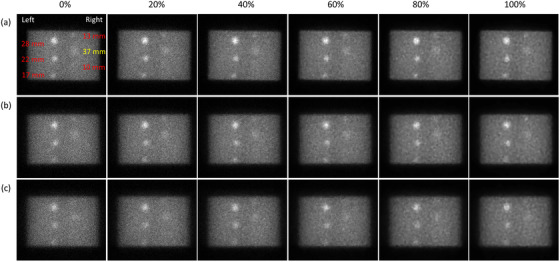
Posterior view of Tc‐99m planar scintigraphy (window level = 50/window width = 100) acquired with a blend ratio of 0, 20, 40, 60, 80, and 100% (from left to right) for (a) NEMA_small_, (b) NEMA_medium_, and (c) NEMA_large_

**FIGURE 5:**
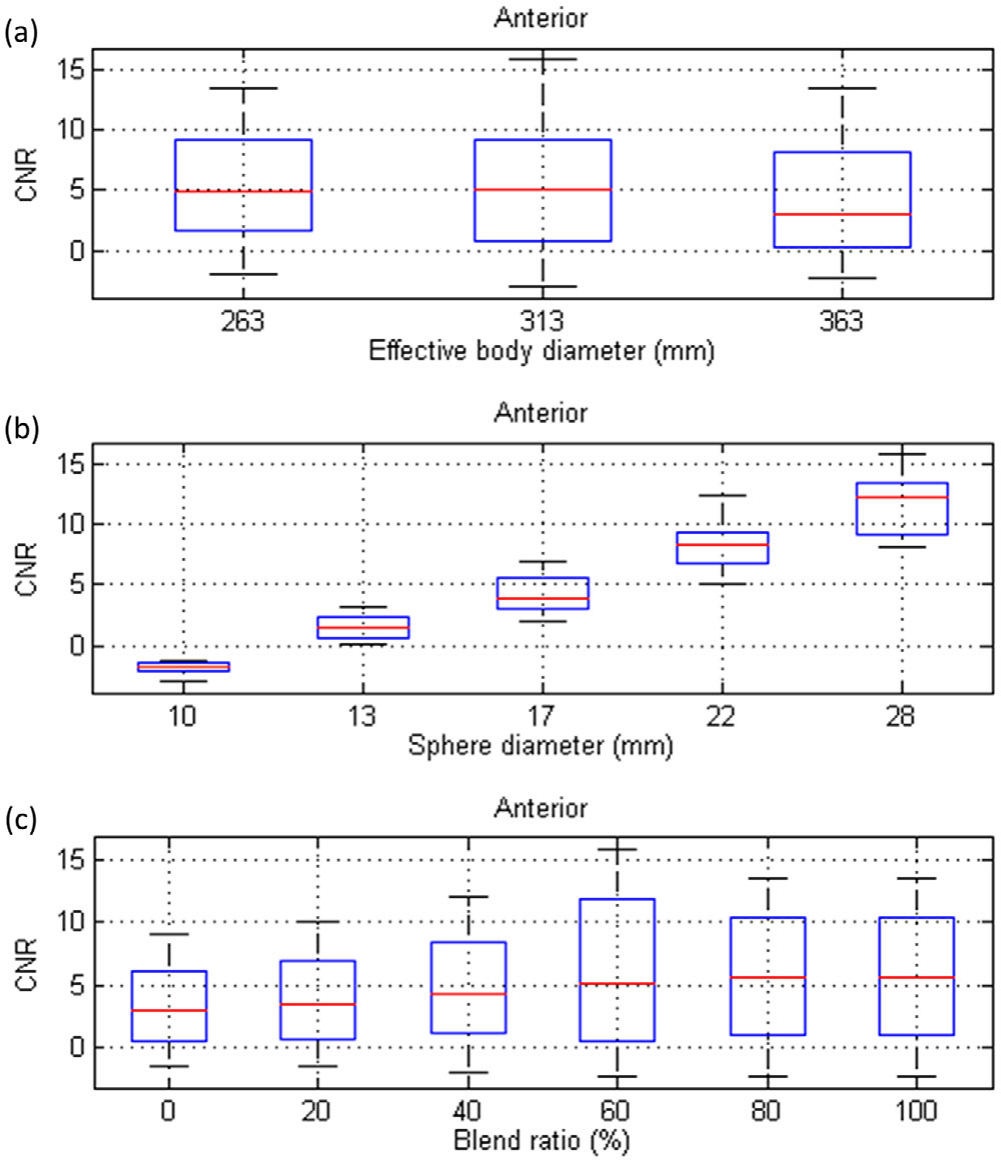
Box and whisker diagrams for contrast‐to‐noise ratio (CNR) in the anterior view of Tc‐99m planar scintigraphy with respective to (a) effect body diameter, (b) sphere diameter, and (c) blend ratio

Figure 3 shows the anterior view of Tc‐99 m planar scintigraphy acquired with an LEHRS collimator and processed with a blend ratio of 0%, 20%, 40%, 60%, 80%, and 100% for a NEMA IEC phantom in three different sizes, whereas Figure 4 shows the corresponding planar scintigraphy in a posterior view. Figure 5 demonstrates the box and whisker diagrams for CNR in the anterior view of Tc‐99m planar scintigraphy shown in Figure 3 with respective to effective body diameter, sphere diameter, and blend ratio. The red line in each box represents the median of the distribution, whereas the top and bottom of each box represent the 25th and 75th percentile of the distribution, respectively. The whiskers extend to the minimum and maximum values for a data set. As seen in Figure 5a, CNR was decreased as the body size increased. On the other hand, CNR shows substantial increase as the sphere diameter increased (see Figure 5b). With regards to the impact of Clarity 2D blending weight, CNR shows mild increase as the blend ratio increased (see Figure 5c). The results of regression analysis of Equation (3) for the anterior planar views shown in Figure 3 were summarized in Table 2. The regression equation that expresses the relationship between CNR and the predictors for Tc‐99m planar scintigraphy acquired with LEHRS collimator and processed with Clarity 2D in anterior view was as follows:

(4) $$\begin{array}{ccc} \;\;\;\;\;\;\;\;\mathrm{CNR} & = & -13.28+954.11\times \left(\frac{1}{\mathrm{body}\;\mathrm{size}}\right)+0.75\\ & & \times \left(\mathrm{target}\;\mathrm{size}\right)+9.57\times (\log(1+\mathrm{blend}\;\mathrm{ratio})) \end{array}$$

**TABLE 2:** Statistical analysis results of the regression model for contrast‐to‐noise ratio (CNR) in anterior planar view

Predictor	B	*β*	*t* ^a^	VIF^b^
1/Effective body diameter	954.1062	0.0813	2.90	1.00
Sphere diameter	0.7451	0.9426	33.65	1.00
Log(1 + blend ratio)	9.5672	0.1934	6.91	1.00

^a^A predictor is considered to be statistically significant if |*t*| > 2.

^b^ A maximum VIF value in excess of 10 is taken as an indication that multicollinearity may be unduly influencing the least square estimates.

**FIGURE 6:**
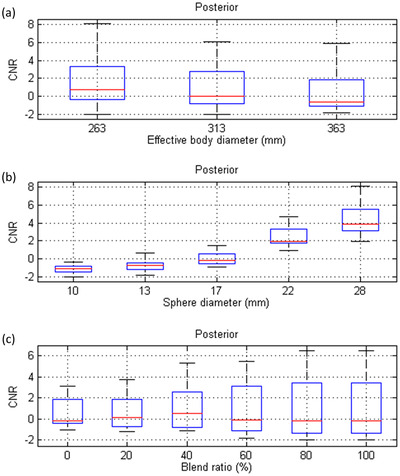
Box and whisker diagrams for contrast‐to‐noise ratio (CNR) in the posterior view of Tc‐99m planar scintigraphy with respective to (a) effect body diameter, (b) sphere diameter, and (c) blend ratio

The regression model in Equation (4) yielded an *R* ^2^ of 0.93. Figure 6 demonstrates the box and whisker diagrams for CNR in the posterior view of Tc‐99m planar scintigraphy shown in Figure 4 with respective to effective body diameter, sphere diameter, and blend ratio. Similar CNR trends have been found in Figure 6 as those in Figure 5. The results of the regression analysis of Equation (3) for the posterior planar views shown in Figure 4 were summarized in Table 3. The regression equation that expresses the relationship between CNR and the predictors for Tc‐99m planar scintigraphy acquired with an LEHRS collimator and processed with Clarity 2D in posterior view was as follows:

(5) $$\begin{array}{ccc} \mathrm{CNR} & = & -8.77+1041.93\times \left(\frac{1}{body\;size}\right)+0.32\times \\ & & \left(\mathrm{target}\;\mathrm{size}\right)+3.37\times (\log(1+\mathrm{blend}\;\mathrm{ratio})) \end{array}$$

**TABLE 3:** Statistical analysis results of the regression model for contrast‐to‐noise ratio (CNR) in posterior planar view

Predictor	B	*β*	*t* ^a^	VIF^b^
1/Effective body diameter	1041.9271	0.1944	4.74	1.00
Sphere diameter	0.3220	0.8919	21.76	1.00
Log(1 + blend ratio)	3.3725	0.1493	3.64	1.00

^a^A predictor is considered to be statistically significant if |*t*| > 2.

^b^A maximum VIF value in excess of 10 is taken as an indication that multicollinearity may be unduly influencing the least square estimates.

**FIGURE 7:**
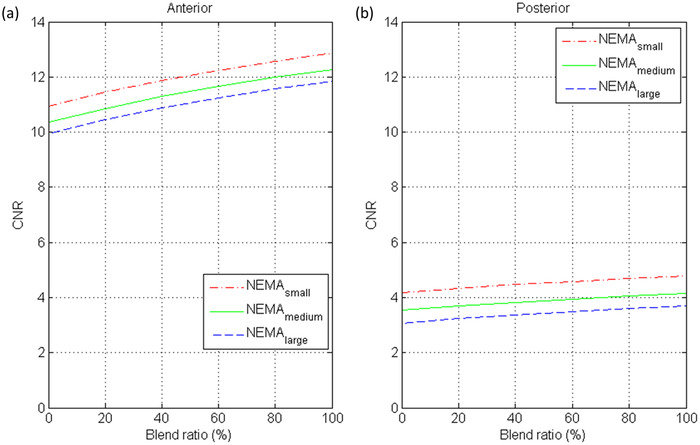
Contrast‐to‐noise ratio (CNR) as a function of blend ratio for 28 mm‐diameter sphere in NEMA_small_, NEMA_medium_, and NEMA_large_ estimated based on the regression models for (a) the anterior view and (b) the posterior view of Tc‐99m planar scintigraphy.

The regression model in Equation (5) yielded an *R* ^2^ of 0.86. Figure 7 demonstrates CNR as a function of blend ratio for 28 mm‐diameter sphere in NEMA_small_, NEMA_medium_, NEMA_large_ estimated based on Equations (4) and (5).

## 4 DISCUSSION

In nuclear medicine imaging, the emitted photons from patient body may undergo photoelectric absorption and Compton scattering. When the number of detected counts is reduced due to photon attenuation, the statistical fluctuation in nuclear medicine imaging is increased consequently.^18^, ^19^ Compton scattering changes the direction in which a primary photon should move, so the scattered photons lead to a background haze in nuclear medicine imaging and thus degrade the image contrast.^20^, ^21^ The magnitude of photon attenuation and scattering is higher in patients with larger body size, so nuclear medicine imaging usually has lower lesion detectability in obese patients.^12^, ^13^ In nuclear medicine imaging, factors affecting image quality can be divided into three categories: (1) the physics of the image formation, (2) the choice of image protocol parameters, (3) biology and physiology of the patient.^22^ Although photon attenuation and scattering are inevitable during image formation, their impacts on image quality can be eased by employing appropriate image protocol parameters.

According to Thibault et al., Tc‐99m planar imaging acquired with an LEHRS collimator and processed by Clarity 2D provides better resolution recovery and noise treatment when compared to that acquired with the conventional LEHR collimator and without further image processing.^10^ This phenomenon was observed in line spread function (LSF) measurements conducted in air and PMMA attenuating medium, whereas the improvement achieved by LEHRS collimator was more obvious in PMMA. In our opinion, this result suggested that the image quality of Tc‐99m planar scintigraphy for obese patients (high attenuation) can be improved by acquiring data with the LEHRS collimator and processed by Clarity 2D. However, there was no other data supporting this hypothesis except LSF, and a blend ratio of 40% was always used in their study. Another study presented by Shibutani et al. has investigated the impact of changing Clarity 2D blending weight on the image quality of gamma scintigraphy acquired with an LEHRS collimator.^11^ They found out that increasing the blend ratio did not affect contrast ratio but can reduce image noise, so CNR was increased when using a higher blend ratio. However, only one phantom size was investigated in their work, so the impact of changing blend ratio for patients with different body sizes was unclear. Bone scintigraphy using Tc‐99m MDP plays a pivotal role for the screening and follow‐up of skeletal metastasis in cancer patients.^23^, ^24^ As cancer treatment may lead to significant body composition changes, it is important to understand the impact of target size and Clarity 2D blending weight on the lesion detectability of Tc‐99m planar scintigraphy acquired with an LEHRS collimator for patients with different body sizes. Therefore, multivariate analysis was used to examine the CNR of Tc‐99m planar scintigraphy acquired under various imaging conditions for NEMA_small_, NEMA_medium_, and NEMA_large_.

Based on naked eye observation, the spheres in NEMA IEC phantom can be detected more easily in Tc‐99m planar scintigraphy that was acquired with a higher blend ratio. However, the noise pattern and target contour were also changed when changing the blend ratio. In order to reduce the biases introduced into CNR calculation due to ROI definition, the workflow shown in Figure 2 was proposed. The regression relationship for either anterior or posterior view has *R* ^2^ larger than 0.80, indicating a good fit to the data. According to our results in Table 2, it was found that all independent variables were statistically significant predictors of CNR in anterior view (|*t*| > 2), whereas the sphere diameter was the most significant predictor (*β* = 0.94), followed by log(1 + blend ratio) (*β* = 0.19) and 1/effective body size (*β* = 0.08). High multicollinearity was not observed among independent variables in the model for anterior view (VIF <10). With regards to the posterior view, all the *t* values shown in Table 3 were larger than 2, indicating the investigated variables were statistically significant to predict CNR. The most significant predictor of CNR in posterior view was a sphere diameter (*β* = 0.89), followed by 1/effective body size (*β* = 0.19) and log(1 + blend ratio) (*β* = 0.15). Again, high multicollinearity was not observed among independent variables in the model for posterior view (VIF <10). The CNR estimated based on Equation (4) as a function of Clarity 2D blending weight was shown in Figure 7a, whereas the CNR estimated based on Equation (5) was shown in Figure 7b. In Figure 7, it was found that changing the blend ratio could improve CNR, and this phenomenon was more significant in anterior view than in posterior view. Furthermore, the estimated CNR shown in Figure 7 also implied that the blend ratio should be selected according to patient body size in order to maintain consistent CNR. Hence, when a blend ratio of 60% was used for a patient before cancer treatment, a lower blend ratio should be used for the same patient experiencing treatment‐related weight loss to achieve consistent lesion detectability in Tc‐99m planar scintigraphy acquired with LEHRS and processed by Clarity 2D.

Several limitations to this study need to be acknowledged. First, the data acquisition and processing procedures investigated in this work are available in a single‐manufacturer's gamma camera systems. Hence, the optimized protocols can only be used on GE gamma cameras equipped with an LEHRS collimator, including Discovery NM/CT 670 and NM/CT 870 DR. Second, this study was conducted by using the NEMA IEC phantom to evaluate the impact of body size on the lesion detectability of Tc‐99m planar scintigraphy. Although NEMA IEC phantom is a phantom recommended by NEMA 2001 Standard for scanner performance characterization,^25^ patient study assessing the efficacy of the optimized protocols on improving the lesion detectability in bone scintigraphy using Tc‐99m MDP will be needed and valuable.

## 5 CONCLUSION

The magnitude of photon attenuation and scattering is higher in patients with larger body size, so nuclear medicine imaging usually has lower lesion detectability in obese patients. Although photon attenuation and scattering are inevitable during image formation, their impacts on image quality can be eased by employing appropriate image protocol parameters. This study investigated the lesion detectability of Tc‐99m planar scintigraphy acquired with LEHRS collimator and processed by Clarity 2D for patients with different body sizes through phantom study. It was found that increasing Clarity 2D blending weight could improve the lesion detectability in Tc‐99m planar scintigraphy, especially in anterior view. Furthermore, Clarity 2D blending weight should be selected according to patient body size in order to maintain consistent lesion detectability. Our study results could be applied for cancer patients in detecting skeletal metastasis based on bone scintigraphy using Tc‐99m MDP.

## CONFLICT OF INTEREST

The authors declare no conflict of interest.

## AUTHOR CONTRIBUTIONS

Conceptualization, KYK and CCY; methodology, CCY; software, CCY; validation, PYL, KJJ and CCY; formal analysis, PYL, KJJ and CCY; investigation, PYL, KJJ and CCY; resources, KYK and CCY; data curation, PYL and CCY; writing, CCY; visualization, CCY; supervision, KYK and CCY; project administration, KYK and CCY.

## References

[1] Banerjee S , Pillai MR , Ramamoorthy N . Evolution of Tc‐99m in diagnostic radiopharmaceuticals. Semin Nucl Med. 2001;31(4):260‐277. 11710769 10.1053/snuc.2001.26205

[2] Dokić DD . Technetium‐99m radiopharmaceuticals for in vivo diagnostics. Med Pregl. 2005;58(3‐4):180‐184. 16526218 10.2298/mpns0504180d

[3] Wrzesień M . Simplicity or complexity of the radiopharmaceutical production process in the light of optimization of radiation protection of staff – 99mTc vs. 18F. Med Pr. 2018;69(3):317‐327. 29790486 10.13075/mp.5893.00687

[4] O'Connor MK , Brown ML , Hung JC , Hayostek RJ . The art of bone scintigraphy–technical aspects. J Nucl Med. 1991;32(12):2332‐2341. 1744725

[5] Van den Wyngaert T , Strobel K , Kampen WU , et al. The EANM practice guidelines for bone scintigraphy. Eur J Nucl Med Mol Imaging. 2016;43(9):1723‐1738. 27262701 10.1007/s00259-016-3415-4 PMC4932135

[6] Van Audenhaege K , Van Holen R , Vandenberghe S , Vanhove C , Metzler SD , Moore SC . Review of SPECT collimator selection, optimization, and fabrication for clinical and preclinical imaging. Med Phys. 2015;42(8):4796‐4813. 26233207 10.1118/1.4927061 PMC5148182

[7] Moore SC , Kouris K , Cullum I . Collimator design for single photon emission tomography. Eur J Nucl Med. 1992;19(2):138‐150. 1563442 10.1007/BF00184130

[8] Zhang B , Zeng GL . High‐resolution versus high‐sensitivity SPECT imaging with geometric blurring compensation for various parallel‐hole collimation geometries. IEEE Trans Inf Technol Biomed. 2010;14(4):1121‐1127. 20460211 10.1109/TITB.2010.2050145 PMC5292234

[9] Kimiaei S , Larsson SA , Jacobsson H . Collimator design for improved spatial resolution in SPECT and planar scintigraphy. J Nucl Med. 1996;37(8):1417‐1421. 8708787

[10] Thibault F , Bailly M , Le Rouzic G , Metrard G . Clinical evaluation of general electric new SwiftScan solution in bone scintigraphy on NAI‐camera: a head to head comparison with Siemens Symbia. PLoS One. 2019;14(9):e0222490. 31536519 10.1371/journal.pone.0222490 PMC6752842

[11] Shibutani T , Onoguchi M , Naoi Y , et al. The usefulness of SwiftScan technology for bone scintigraphy using a novel anthropomorphic phantom. Sci Rep. 2021;11(1):2644. 33514818 10.1038/s41598-021-82082-x PMC7846574

[12] Hansen CL , Woodhouse S , Kramer M . Effect of patient obesity on the accuracy of thallium‐201 myocardial perfusion imaging. Am J Cardiol. 2000;85(6):749‐752. 12000052 10.1016/s0002-9149(99)00853-x

[13] Ghanem MA , Kazim NA , Elgazzar AH . Impact of obesity on nuclear medicine imaging. J Nucl Med Technol. 2011;39(1):40‐50. 21321247 10.2967/jnmt.110.078881

[14] Elad M . On the origin of the bilateral filter and ways to improve it. IEEE Trans Image Process. 2002;11(10):1141‐1151. 18249686 10.1109/TIP.2002.801126

[15] Winkler G , Aurich V , Hahn K , Martin A , Rodenacker K . Noise reduction in images: some recent edge‐preserving methods. J Pattern Recognit Image Anal. 1999;9:749‐766.

[16] Richardson WH . Bayesian‐based iterative method of image restoration. J Opt Soc Am B: Opt Phys. 1972;62:55‐59.

[17] Lucy LB . An iterative technique for the rectification of observed distributions. Astron J. 1974;79:745.

[18] Tsui BM , Zhao X , Frey EC , McCartney WH . Quantitative single‐photon emission computed tomography: basics and clinical considerations. Semin Nucl Med. 1994;24(1):38‐65. 8122128 10.1016/s0001-2998(05)80248-x

[19] Patton JA , Turkington TG . SPECT/CT physical principles and attenuation correction. J Nucl Med Technol. 2008;36(1):1‐10. 18287196 10.2967/jnmt.107.046839

[20] Hutton BF , Buvat I , Beekman FJ . Review and current status of SPECT scatter correction. Phys Med Biol. 2011;56(14):R85‐112. 21701055 10.1088/0031-9155/56/14/R01

[21] Buvat I , Benali H , Todd‐Pokropek A , Di Paola R . Scatter correction in scintigraphy: the state of the art. Eur J Nucl Med. 1994;21(7):675‐694. 7957356 10.1007/BF00285592

[22] Frey EC , Humm JL , Ljungberg M . Accuracy and precision of radioactivity quantification in nuclear medicine images. Semin Nucl Med. 2012;42(3):208‐218. 22475429 10.1053/j.semnuclmed.2011.11.003 PMC3586419

[23] Love C , Din AS , Tomas MB , Kalapparambath TP , Palestro CJ . Radionuclide bone imaging: an illustrative review. Radiographics. 2003;23(2):341‐358. 12640151 10.1148/rg.232025103

[24] Gold RI , Seeger LL , Bassett LW , Steckel RJ . An integrated approach to the evaluation of metastatic bone disease. Radiol Clin North Am. 1990;28(2):471‐483. 2408106

[25] Association NEM . Performance Measurements of Scintillation Cameras. National Electrical Manufacturers Association; 2001.

